# Memokath for treating ureteric stricture post cryoablation of renal mass: A case report of rare complication and proposed alternative management

**DOI:** 10.1016/j.radcr.2022.10.033

**Published:** 2022-11-09

**Authors:** Ibrahim A. Khalil, Nagy Younes, khalid Awad, Maya Aldeeb, Omar M. Aboumarzouk, Khalid Al-Rumaihi, Abdulla Al-Ansari

**Affiliations:** a Department of Urology, Hamad Medical Corporation, PO Box 3050, Doha, Qatar; b Department of Medical Education, Family Medicine Residency Program, Hamad Medical Corporation, Doha, Qatar; c Department of Surgery, Surgical Research Section, Hamad Medical Hospital, Hamad Medical Corporation, Doha, Qatar; d College of Medicine, Qatar University, Doha, Qatar; e The University of Medicine, Veterinary and Life Science, University of Glasgow, Scotland, UK

**Keywords:** Memokath, Cryoablation, Ureteral stricture, Small renal mass, Iatrogenic stricture, Case report

## Abstract

The use of cryoablation in the management of small renal masses is widely acceptable. Although rare but ureteral injury during the procedure with subsequent stricture formation can result in devastating effects on renal function. On the other hand, the management of such strictures requires reconstructive surgery as gold standard. Unfortunately, in some cases the reconstructive surgery might not be feasible, and the treatment usually is ureteral stent insertion that need to be changed regularly. Here we present a case of a 53-year-old gentleman who developed an upper ureteric iatrogenic stricture post cryoablation in which the reconstructive surgery was not feasible due to high procedural risk. We used metallic ureteral stent (Memokath) instead of regular ureteral double J stent. We found that if the reconstructive surgery is not possible the usage of Memokath in treating iatrogenic ureteral strictures is associated with better quality of life, lower costs and a similar functional outcome when compared to ureteral double J stent that needs regular frequent changes.

## Introduction

With the ubiquitous use of cross-sectional abdominal imaging in recent years, the incidence of small renal masses (SRMs) has increased, and the evaluation and management of SRMs have become critical clinical issues. In patients with bilateral renal masses, the management is more challenging as a maximum reservation of renal function is required. Different options for nephron-sparing interventions are available in guidelines as partial nephrectomy and ablative treatment, such as radiofrequency ablation (RFA) and cryoablation [1,2]. Studies showed that cryoablation has a better complication profile when compared to RFA. The Iatrogenic development of upper ureteric stricture and pelvicalyceal system injury is up to 25% in RFA compared to cryoablation, as only one case iatrogenic stricture post cryoablation reported in literature [3,4]. The definitive treatment of iatrogenic upper ureteric stricture is ureteral reconstructive, but the challenge is when the reconstructive surgery cannot be done, which necessitates drainage by a regularly changed ureteral stent or nephrostomy tube insertion, both of which can result in significant effects on quality of life. Here we presented a case of iatrogenic upper ureteric stricture post cryoablation in which reconstructive surgery was not feasible treated with retrograde insertion of thermosensitive tightly coiled metallic stent (Memokath) as the first report in literature and proposed alternative management.

This case report has been reported in line with the SCARE Criteria [5].

## Case report

### Case presentation

**Fig. 1:**
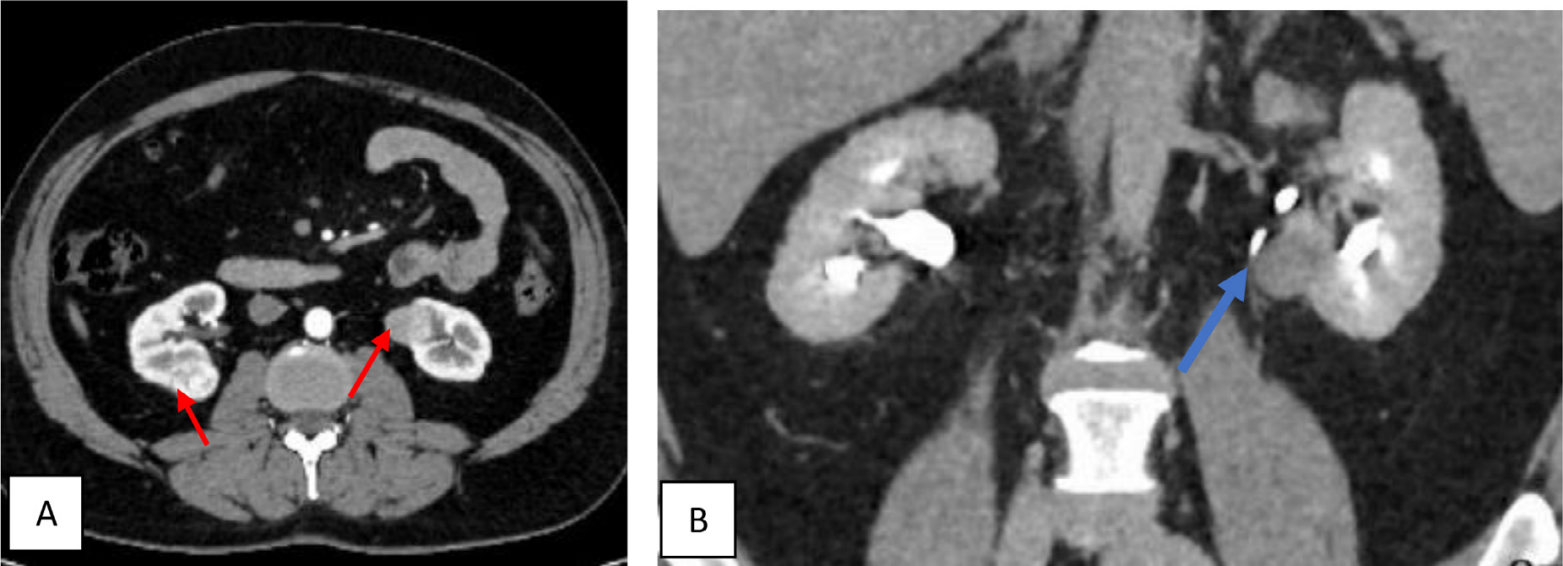
CT scan showing (A): bilateral small renal masses in axial section s (red arrows), (B) left renal mass in lower pole with proximity to left upper ureter (blue arrow).

**Fig. 2:**
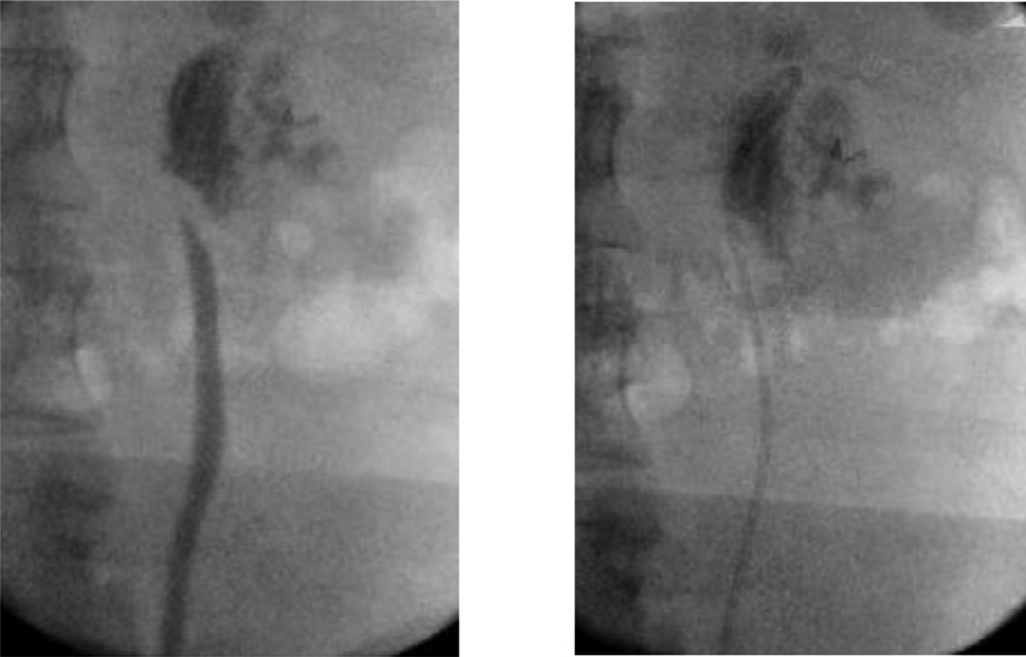
Retrograde pyelogram showing left upper ureteric stricture and passed by guidewire and double J inserted.

**Fig. 3:**
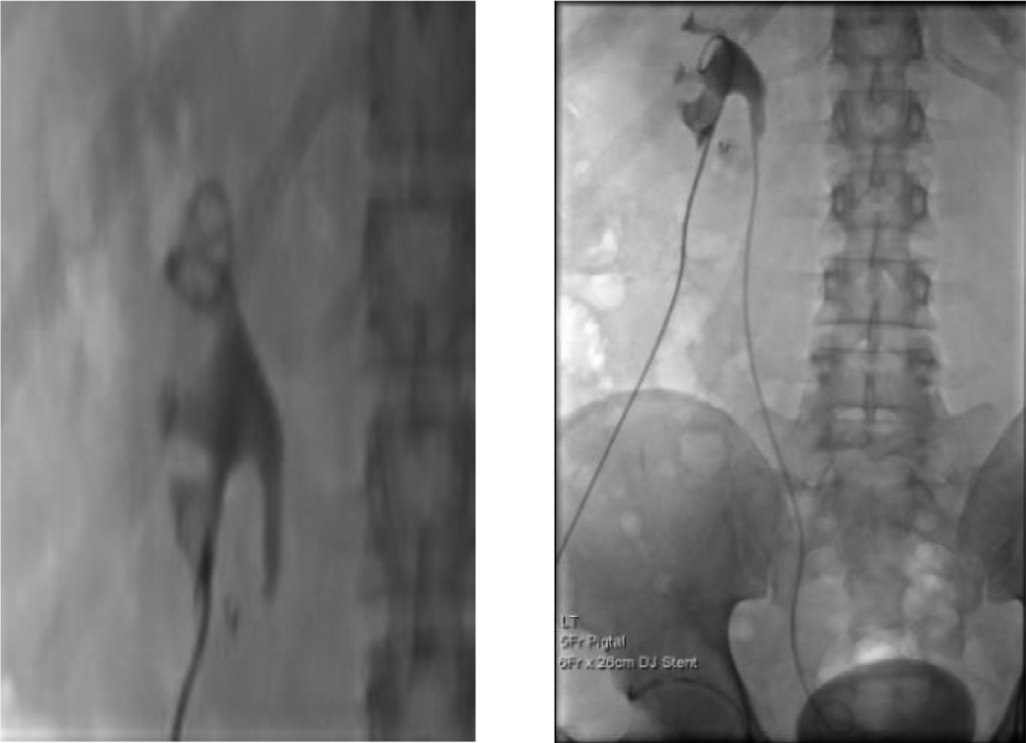
Nephrostogram showing obliteration of left upper ureter and antegrade DJ stent insertion.

**Fig. 4:**
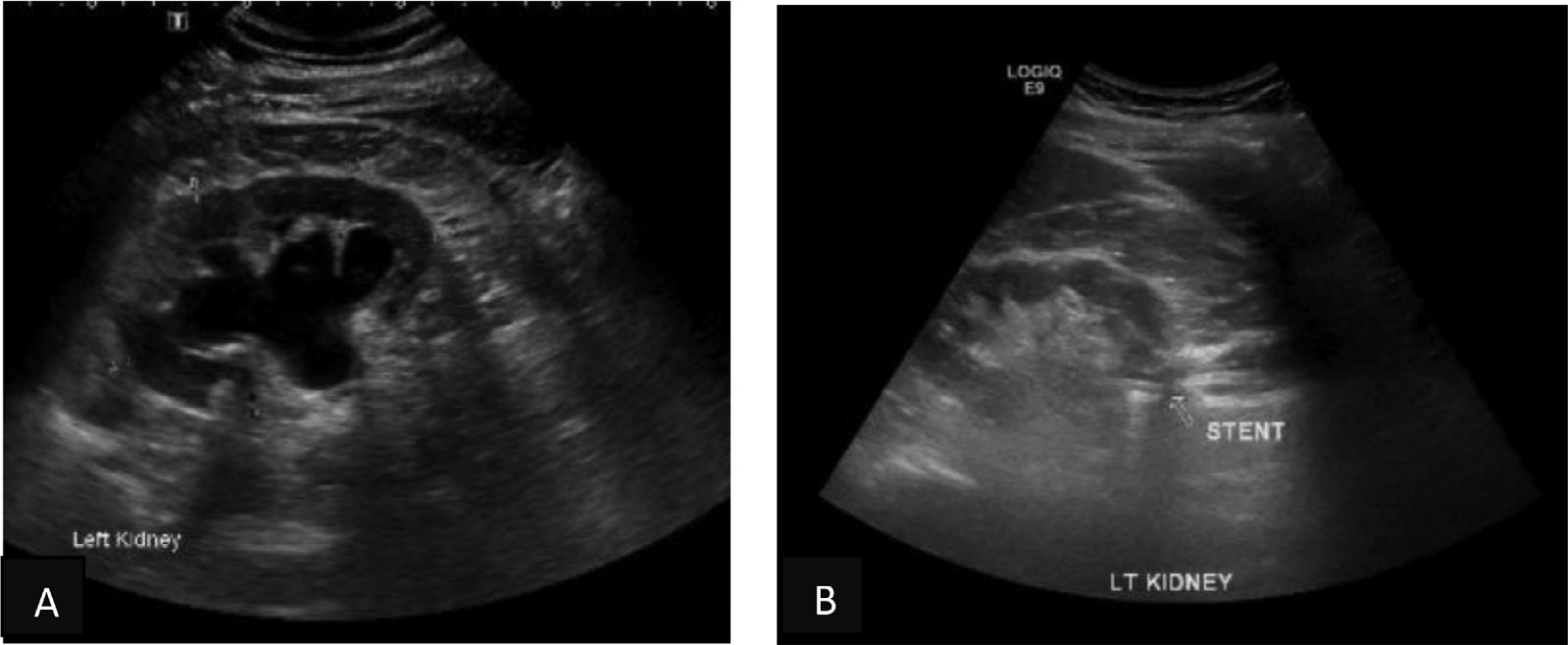
Ultrasound of left kidney, (A) hydronephrosis, (B) after Memokath insertion resolution of hydronephrosis.

**Fig. 5:**
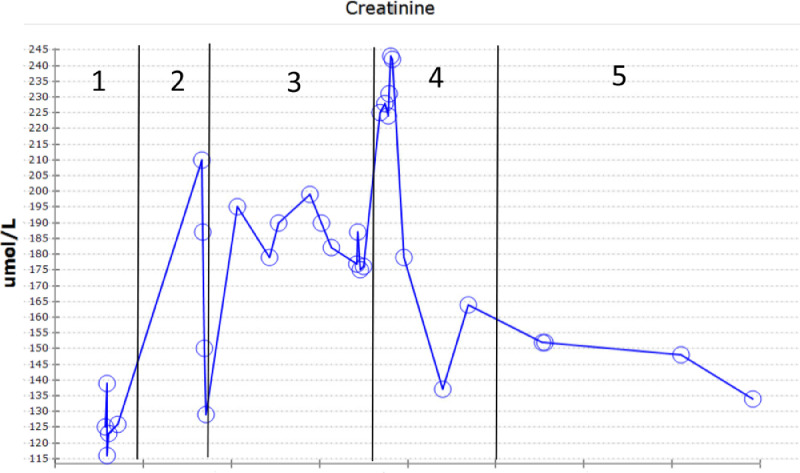
Renal function tracking, 1: cryoablation, 2: post cryoablation, 3: drainage by double J, 4: nephrostomy tube and antegrade stent insertion, 5; Memokath insertion.

A 53-year-old gentleman, medically free and has no previous surgeries. Patient was found to have a bilateral renal mass on computed topography scan (CT) done for abdominal pain (Fig. 1). The patient underwent cryoablation and biopsy for the right renal mass, followed by cryoablation and biopsy of the left renal mass; biopsies showed clear cell carcinoma bilaterally. Two months after cryoablation of the left renal mass patient started to complain of left flank pain associated with worsening renal function. CT abdomen showed left hydronephrosis, retrograde pyelogram showed iatrogenic upper ureteric stricture (Fig. 2), primarily due to proximity of the ablated tumor to the upper ureter, for which double J stent (DJ) inserted retrogradely. Follow-up ultrasound (US) showed the DJ in the upper ureter, so a left nephrostomy tube was inserted, nephrostogram showed no passage of contrast from the renal pelvis to the upper ureter, confirming the diagnosis of upper ureteric stricture and a DJ stent was inserted antegradely with subsequent resolution of pain and improvement in renal function Fig. 3. The case was discussed in uro-oncology multiple disciplinary team and reviewed by 3 reconstructive surgeons; all reported complex surgical procedures with high failure rates. Then, after discussion with the patient the available options as regular DJ change versus Memokath insertion. The patient preferred and underwent balloon dilatation with Memokath insertion. Upon follow-up, 6 month after Memokath insertion, the patient was asymptomatic, hydronephrosis resolved (Fig. 4) and renal function improved (Fig. 5). Also, there were no complications or adverse outcomes.

## Procedures description

### Cryoablation

**Fig. 6:**
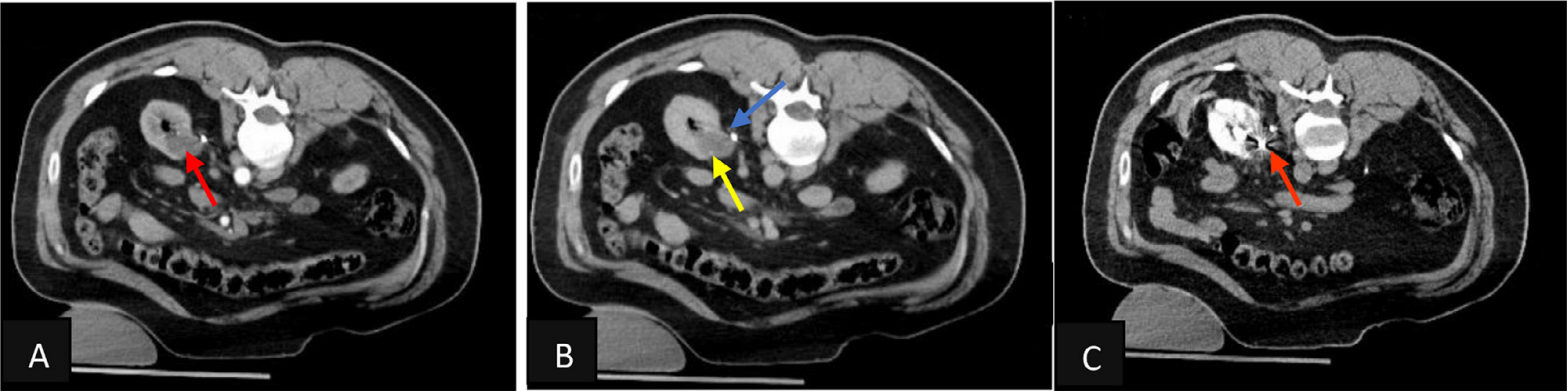
Triphasic CT scan during cryoablation procedure (A) showing the tumor in anterior lower calyx of left kidney in arterial phase (red arrow), (B) delayed excretory phase showing the close proximity of the tumor (yellow arrow) to the ureter (blue arrow), (C) the intraprocedural CT scan showing cryoablation of the tumor with possible involvement of the upper ureter (orange arrow).

The procedure performed in the prone position under general anesthesia using an angio-CT suite after retrograde insertion of ureteric DJ stent. Planning triphasic CT scan performed to outline the lesion, core biopsy taken from the left kidney anterior lower pole lesion using coaxial system 18G x 16cm biopsy needle. Additionally, a coil marker was placed into the lesion through already placed co-axial needle. Three 16G cryoablation needles were then placed into the left renal lesion. Cryoablation using Argon Gas was started for 10 minutes followed by passive thawing until temperature reached 0 which took almost 6 minutes followed by a 2nd cycle of Cryoablation for 10 minutes followed by active thawing using Helium Gas until temperature reached 30 Celsius and then the needles were removed (Fig. 6).

### Ureteral dilatation and Memokath insertion

**Fig. 7:**
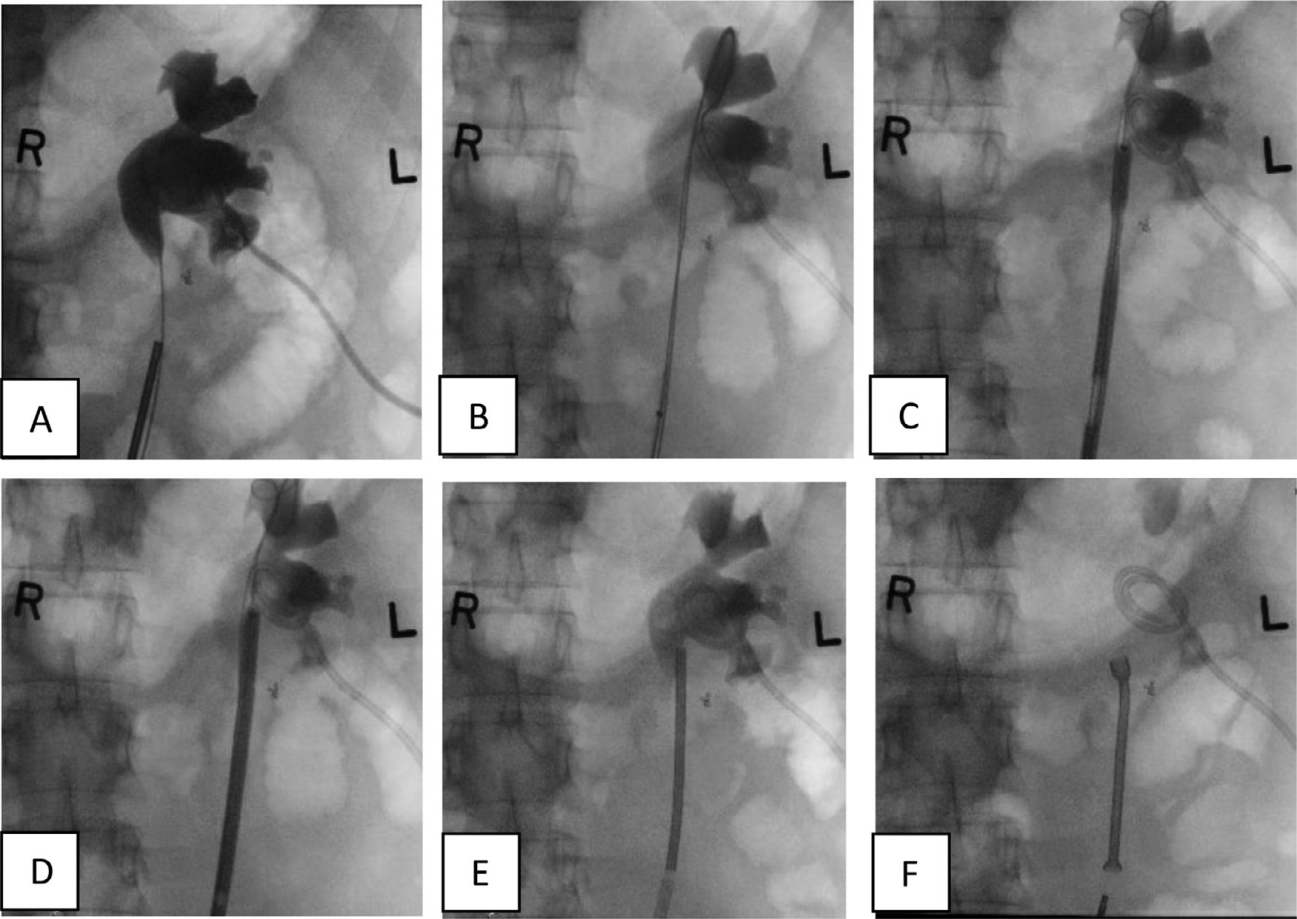
Intraoperative X-rays showing: Nephrostogram with upper ureteric blockage (A) bypass of the left upper ureteric stricture with guidewire and balloon dilatation (B-D). Memokath insertion in left upper ureter (E,F).

Under general anesthesia and in lithotomy position, cystoscopy was unremarkable, then we cannulated the left ureter using sensor guidewire and a 5F open ended catheter and did retrograde pyelography that showed complete blockage of the upper ureter and contrast is not reaching the kidney. Then we did Left Nephrostogram showed dilated system, with no passage of contrast into the ureter. We used second sensor guide wire to bypass the upper ureteric stricture, followed by Balloon dilatation and fixation of Memokath (Fig. 7A-F).

## Literature review

A search of the PubMed database was undertaken on 01/08/2022 with the search terms ((((ureteral) OR (ureteric)) AND (stricture)) OR (Narrowing)) AND (cryoablation) *Filters applied: in the last 10 years, Humans, English*. The search yielded 24 results of which only one paper was identified as report of iatrogenic ureteric stricture post image guided renal cryoablation [4].

## Discussion

Treatment options for small renal mass include nephron-sparing surgeries, ablative treatment, and active surveillance [1,2]. The use of cryoablation mainly in patients with renal masses related to syndromes like von Hippel Lindau (VHL) syndrome or bilateral renal tumor as the risk of recurrent disease and the need for multiple interventions necessitate treatment with the most negligible effect on renal function. Ablation of small renal masses is associated with complications like; hemorrhage, vascular injury, and urothelial injury resulting in urine leak or stricture [6]. The stricture due to thermal injury is higher in radiofrequency ablation than in cryoablation, in which the stricture formation is very rare with one case report in literature [3,4,7]. The cause of ureteric stricture is the involvement of the upper ureter with the cryoablation created during the freezing-thawing cycles of cryoablation [8].

Different measures have been proposed to protect the pelvicalyceal system during ablative treatment (radiofrequency or cryoablation) of tumors in close proximity like pyeloperfusion, either ante or retrograde, and hydrodistension [8,9,10]. In our report, the patient has bilateral renal tumors and underwent cryoablation for both; the cryoablation of the left renal mass was complicated by upper ureteric stricture primarily due to the proximity between the renal mass and upper ureter, resulting in the involvement of the upper ureter in the cryoablation ball.

The subsequent development of obstruction due to stricture with back pressure changes and derangement of renal function necessitate drainage of the collecting system either by nephrostomy tube or ureteral stent as temporary measurement, waiting for definitive ureteric reconstruction if possible. In our case, due to cryoablation related adhesions and relatively long stricture, the reconstructive surgery has high procedural risks and failure rates, as reviewed and discussed with different reconstructive surgeons, all of which favored the use of ureteral stent as DJ or Memokath [11]. Literature reported the use of ureteral DJ stent with regular change to treat post cryoablation strictures whenever the surgical intervention is not feasible. In our case, we used an endoscopically inserted ureteral Memokath stent to relieve the obstruction for the first time in such cases.

Memokath is a thermo-expandable, nickel-titanium alloy spiral stent used to treat ureteric obstruction resulting from malignant or benign strictures [12]. The indication of Memokath includes malignant and benign ureteric strictures in which reconstructive surgeries are not feasible due to failure risks or patient preference [11]. The clinical success rate of Memokath is comparable to the use of a double J stent, with a 100% success rate [13]. On the other hand, Memokath is more cost-effective than regularly changed double J stent [14]. Furthermore, Memokath has no stent irritation effect, as seen in the Double J stent. Memokath is certified MRI safe, which is of great importance, especially in a patient with renal masses in which regular follow-up imaging is required to detect the development of recurrences or progression [15].

Here, we report a rare case of iatrogenic upper ureteric stricture due to cryoablation of renal mass in close proximity with the ureter treated by endoscopic insertion of Memokath for the first time in literature resulting in the preservation of renal function and resolution of patient's symptoms and hydronephrosis with better quality of life and lower cost on long run.

## Conclusion

Iatrogenic upper ureteric stricture is a rare complication of cryoablation or renal masses. The gold standard for treatment is reconstructive surgery. In cases in which the ureteric reconstructive surgery is not possible we propose the use of ureteral Memokath in treating obstruction as a better alternative to double J stent with lower cost, fewer side effects and similar functional outcomes.

## Patient consent

Informed consent was obtained from the patient according to our hospital policy.
